# Fbxw7 suppresses carcinogenesis and stemness in triple-negative breast cancer through CHD4 degradation and Wnt/β-catenin pathway inhibition

**DOI:** 10.1186/s12967-024-04897-2

**Published:** 2024-01-24

**Authors:** Guodong Xiao, Weiping Lu, Jing Yuan, Zuyue Liu, Peili Wang, Huijie Fan

**Affiliations:** 1 Department of Oncology, The First Affiliated Hospital of Zhengzhou University, No.1 Jianshe Dong Road, ErQi District, Zhengzhou, 450052 Henan China; 2 Breast Cancer Center, Affiliated Cancer Hospital of Zhengzhou University, Henan Cancer Hospital, No 127 Dongming Road, Jinshui District, Zhengzhou, 450008 Henan China

**Keywords:** Fbxw7, CHD4, Stemness, Wnt/β-catenin, Triple-negative breast cancer

## Abstract

**Background:**

Cancer stem cells (CSCs) are a small population of cells in tumor tissues that can drive tumor initiation and promote tumor progression. A small number of previous studies indirectly mentioned the role of F-box and WD repeat domain-containing 7 (FBXW7) as a tumor suppressor in Triple-negative breast cancer (TNBC). However, few studies have focused on the function of FBXW7 in cancer stemness in TNBC and the related mechanism.

**Methods:**

We detected FBXW7 by immunohistochemistry (IHC) in 80 TNBC patients. FBXW7 knockdown and overexpression in MD-MBA-231 and HCC1937 cell models were constructed. The effect of FBXW7 on malignant phenotype and stemness was assessed by colony assays, flow cytometry, transwell assays, western blot, and sphere formation assays. Immunoprecipitation-Mass Spectrometry (IP-MS) and ubiquitination experiments were used to find and verify potential downstream substrate proteins of FBXW7. Animal experiments were constructed to examine the effect of FBXW7 on tumorigenic potential and cancer stemness of TNBC cells in vivo.

**Results:**

The results showed that FBXW7 was expressed at low levels in TNBC tissues and positively correlated with prognosis of TNBC patients. In vitro, FBXW7 significantly inhibited colony formation, cell cycle progression, cell migration, EMT process, cancer stemness and promotes apoptosis. Further experiments confirmed that chromodomain-helicase-DNA-binding protein 4 (CHD4) is a novel downstream target of FBXW7 and is downregulated by FBXW7 via proteasomal degradation. Moreover, CHD4 could promote the nuclear translocation of β-catenin and reverse the inhibitory effect of FBXW7 on β-catenin, and ultimately activate the Wnt/β-catenin pathway. Rescue experiments confirmed that the FBXW7-CHD4-Wnt/β-catenin axis was involved in regulating the maintenance of CSC in TNBC cells. In animal experiments, FBXW7 reduced CSC marker expression and suppressed TNBC cell tumorigenesis in vivo.

**Conclusions:**

Taken together, these results highlight that FBXW7 degrades CHD4 protein through ubiquitination, thereby blocking the activation of the Wnt/β-catenin pathway to inhibit the stemness of TNBC cells. Thus, targeting FBXW7 may be a promising strategy for therapeutic intervention against TNBC.

**Supplementary Information:**

The online version contains supplementary material available at 10.1186/s12967-024-04897-2.

## Introduction

Triple-negative breast cancer (TNBC) is characterized by the lack of three key molecular signatures: estrogen receptor (ER) expression, progesterone receptor (PR) expression, and HER2 overexpression; it comprises approximately 15% of all invasive breast cancers [1]. Given the lack of well-defined molecular targets, TNBC has the worst prognosis and fewest treatment options among all breast cancer types. TNBCs also contain a high proportion of breast cancer stem cells (BCSCs) and exhibit chemoresistance [2]. Therefore, the exploration of novel and effective molecular targets to prevent and ultimately cure TNBC remains a critical unmet need.

It is well known that a small population of tumor cells with stem cell-like properties (stemness)—called cancer stem cells (CSCs)—exist within tumors. These CSCs are capable of self-renewal, pluripotent differentiation, metastatic dissemination, and therapeutic resistance. Based on cell surface markers and stem cell-like features, CSCs have been isolated and identified from numerous solid malignancies, including breast cancer [3]. Accumulating evidence indicates that CSCs are responsible for drug resistance, metastasis, and tumor recurrence. In TNBC, BCSCs are commonly characterized by different markers, such as CD44/CD24, ALDH1, CD133, and EPCAM [4]. In addition, the maintenance of BCSC stemness is typically driven by aberrant activation of transcription factors, such as OCT4, SOX2, and BMI1, together with aberrant activation of key signaling pathways, including Wnt, Notch, Hedgehog, and Hippo [5]. Therefore, targeting BCSCs in TNBC may be a crucial strategy to achieve effective treatment.

F-box and WD repeat domain-containing 7 (FBXW7), also known as CDC4, is a substrate recognition component of SKP1-CUL1-F-box (SCF) E3 ubiquitin ligase. Given that most FBXW7 substrates are well-characterized oncoproteins, FBXW7 may serve as a putative tumor suppressor [6]. Consistent with this notion, genomic deletion or mutation of FBXW7 has frequently been reported in various cancers, with a cancer-wide average frequency of 6%. Notably, almost half of the mutations are missense mutations in key arginine residues (Arg 465 and Arg 479) within the substrate recognition site [7]. In our previous study, we showed that FBXW7 inhibits EMT and chemoresistance in NSCLC by regulating the ubiquitination and degradation of Snail [8]. Subsequently, similar observations have been reported for other malignancies [9]. To date, FBXW7 has been reported to regulate cell proliferation, cell migration, cell cycle arrest and apoptosis. However, its function and mechanism in regulating CSCs, especially BCSCs, have not yet been elucidated in TNBC.

Epigenetic regulators that mediate chromatin DNA or histone modifications may be involved in regulating BC heterogeneity, plasticity and tumorigenesis. Identifying the transacting factors and relevant pathways could help to reveal potential therapeutic targets for antitumor therapy. One such epigenetic factor, chromodomain-helicase-DNA-binding protein 4 (CHD4), is a core ATPase subunit of the nucleosome remodeling and deacetylase (NuRD) complex and contributes to protecting genome integrity by controlling the cell cycle and repairing DNA damage [10]. CHD4 is also known to be involved in regulating transcriptional events during tumorigenesis and malignant progression [11]. It has been confirmed to be closely associated with cancer stemness in hepatocellular carcinoma [12], papillary thyroid carcinoma [13] and endometrial cancer [14]. For instance, CHD4 depletion and CHD4 mutations promote endometrial cancer stemness by activating TGF-beta signaling [14]. Interestingly, CHD4 could enhance the metastatic ability and drug resistance of TNBC cells and is also a prognostic marker for TNBC patients [15]. However, whether CHD4 plays an important role in mediating the stemness characteristics of BCSCs in TNBC is still poorly investigated.

Herein, we presented data highlighting a favorable prognostic factor—FBXW7—in TNBC patients and demonstrated that it could be a stemness marker of TNBC in vivo and in vitro. We also found that CHD4 is a potential downstream target of FBXW7, and the enhanced FBXW7 expression significantly suppresses the stemness properties of TNBC cells by facilitating ubiquitin-mediated degradation of CHD4 protein and then affecting the Wnt/β-Catenin pathway. Together, these findings show that FBXW7 physically interacts with CHD4 and mediates its ubiquitination, which highlights that targeting CHD4 may be a promising therapeutic strategy for eradicating BCSCs to overcome tumor relapse, metastasis and drug resistance in TNBC.

## Methods and materials

### Immunohistochemistry (IHC) staining

Human tissue microarray slides containing a total of 80 pairs of TNBC tumor tissues and matched adjacent tissues were purchased from Superbiotek, Inc. (BRC1601, Shanghai, China). Paraffin-embedded tumors were sliced into 6 μm thick sections. The tissue array slides were subjected to standard IHC using protein-specific antibodies as indicated: anti-FBXW7 antibody (1:300, Cat#ab105752, Abcam, USA) and anti-CHD4 (1:200, Cat#ab105752, Abcam, USA). The detailed clinicopathological characteristics are described in Table 1.

**Table 1:** The relationship between the expression of FBXW7 and the clinicopathological characteristics of TNBC patients

FBXW7	number of cases (n = 80)	Low (n = 40)	High (n = 40)	p
Age				0.822572
> 50 years	37	18	19	
≤ 50 years	43	22	21	
Tumor size (cm)				0.498962
> 2	43	24	21	
≤ 2	37	16	19	
TNM				0.807006
I–II	44	27	27	
III–IV	26	13	13	
Lymph node				0.073278
Negative	42	25	17	
Positive	38	15	23	
Survival status				0.00022
Live	50	17	33	
Death	30	23	7	

All data are shown as numbers. Associations of FBXW7 expression levels with clinical characteristics were evaluated using Pearson’s χ^2^ test, Statistical significance was set at p < 0.05 for all analyses

### Cell culture

Human mammary epithelial cells (MCF-10A), two non-TNBC cell lines (MCF-7 and T47D), and three TNBC cell lines (HCC1937, BT-549 and MD-MBA-231) were purchased from the Cell Bank of the Chinese Scientific Academy. MCF-10A cells were cultured in mammary epithelial cell growth medium (MEGM; Bulletkit, Lonza). MCF-7 cells were cultured in minimum essential medium (HyClone, Logan, UT, USA). BT549, T47D, and HCC1937 cells were cultured in RPMI-1640 medium (HyClone, Logan, UT, USA). MD-MBA-231 cells were cultured in DMEM. All of the above-listed media were supplemented with 10% fetal bovine serum (FBS) (BI, Beit-HaEmek, Israel) and 1% penicillin/streptomycin (Beyotime, Shanghai, China). For the mammosphere formation assay, cells were cultured in mammosphere medium containing DMEM/F12 supplemented with 5 mg/ml insulin (Sigma, St. Louis, USA), 2% B27 (Invitrogen, Carlsbad, CA, USA), 20 ng/ml EGF (Invitrogen, Carlsbad, CA, USA) and 20 ng/ml bFGF (Invitrogen, Carlsbad, CA, USA). All cells were cultured in a humidified incubator with an atmosphere of 5% CO2 at 37 °C.

### Chemical reagents

MSAB (10 μM, MCE, HY-120697) was used to inhibit Wnt/β-catenin signaling. LiCl (10 mM, MCE, HY-W094474) was used to activate Wnt/β-catenin signaling. Cycloheximide (10 μg/ml, MCE, HY-12320) was used as a protein synthesis inhibitor. MG132 (MCE, 20 μM, HY-13259) was used as a proteasomal degradation inhibitor.

### Quantitative real-time PCR (qRT-PCR)

Total RNA was isolated from cells using TRIzol reagent (Beyotime, Shanghai, China) as previously described. Reverse transcription and qRT‒PCR were performed according to methods described previously. Following the standard protocol of the PrimeScript™ RT Reagent Kit (Takara, Dalian, China), reverse transcription was conducted to generate cDNA. qRT‒PCR analysis was carried out using SYBR-Green PCR master mix (Takara, Dalian, China). Finally, relative mRNA levels of FBXW7 were calculated with normalization to the reference gene GAPDH mRNA by using the 2^−ΔΔCq^ method. The sequences of primers used for qRT‒PCR analysis are listed in Table 2.

**Table 2:** Human primer sequences used for qRT-PCR

Primers	Forward(5′-3′)	Reverse (5′-3′)
FBXW7	CACTCAAAGTGTGGAATGCAGAGAC	GCATCTCGAGAACCGCTAACAA
CHD4	GGTTTTGGTTCCAAGCGTAA	CTCCTCCTCGCCTTTCTTTT
GAPDH	CGGAGTCAACGGATTTGGTCGTAT	AGCCTTCTCCATGGTGAAGAC

### Western blot analysis and immunoprecipitation

Total protein was extracted from the treated cells by using radioimmunoprecipitation (RIPA) lysis buffer. The extracted total protein was then used in subsequent Western blot experiments as described previously. Sample proteins were separated by electrophoresis on 10% sodium-dodecyl sulfate polyacrylamide gels (SDS‒PAGE) and transferred to a polyvinylidene difluoride (PVDF) membrane. The membrane was blocked with 5% bovine serum albumin (BSA) for 1 h at room temperature. The PVDF membrane was then incubated with primary antibodies overnight at 4 ℃. Antibodies used were anti-Flag antibody (1:1000, Cat#F1804, Sigma), anti-HA antibody (1:1000, Cat#H3663, Sigma), anti-FBXW7 antibody (1:1000, Cat#ab105752, Abcam, USA), anti-CHD4 antibody (1:1000, Cat#ab105752, Abcam, USA), anti-E-cadherin antibody (1:1000, Cat#3195, CST, USA), anti-N-cadherin antibody (1:1000, Cat #13116, CST, USA), anti-Snail1 antibody (1:1000, Cat #3879, CST, USA), anti-Vimentin antibody (1:1000, Cat #5741, CST, USA), anti-SOX2 antibody (1:1000, Cat#ab92494, Abcam, USA), anti-SOX2 antibody (1:1000, Cat#ab92494, Abcam, USA), anti-OCT4 antibody (1:1000, Cat# ab200834, Abcam, USA), anti-NANOG antibody (1:1000, Cat# ab109250, Abcam, USA), anti-EpCAM antibody (1:1000, Cat# ab213500, Abcam, USA), anti-P84 antibody (1:1000, Cat# ab54370, Abcam, USA) and anti-GAPDH antibody (1:1000, Cat#AC026, ABclonal, USA). Subsequently, the PVDF membrane was incubated with horseradish peroxidase-labeled secondary antibodies for 1 h at room temperature. The secondary antibodies for Western blotting were goat anti-mouse IgG (1:5000, Cat#A0216, Beyotime, China) or goat anti-rabbit IgG (1:5000, Cat#A0208, Beyotime, China). Finally, the bands were visualized with an ECL detection system (GE Healthcare). For immunoprecipitation experiments, cells were harvested and lysed using ice-cold NP-40 lysis buffer (Beyotime, Shanghai, China) with protease inhibitors on ice for 30 min. The supernatant of each sample was incubated with 1 μg of anti-FBXW7 or anti-CHD4 at 4 °C overnight. A nonspecific anti-human IgG antibody was used as the negative control. After gentle rocking at 4 °C overnight, protein A/G agarose (#sc-2003, Santa Cruz Biotechnology, USA) was added to the incubated mix and maintained for 4 h at 4 °C to precipitate the antibody-protein complexes. The beads were subsequently collected and washed with IP buffer to collect the supernatant for subsequent Western blot analysis.

### Transfection and infection experiments and plasmids

Specific small hairpin RNAs (shRNAs) targeting FBXW7 and negative control shRNA (sh-NC) were purchased from Gene Pharma Company (Shanghai, China) and cloned and inserted into the pLKO.1-GFP vector to obtain PLKO‐shFBXW7. Full-length human HA-tagged FBXW7 and ΔFbox deletion HA-tagged FBXW7 were cloned and inserted into the lentiviral vector pCDH (System Biosciences, Palo Alto, CA). Full-length FLAG-tagged CHD4 and ΔTPTPS internal deletion FLAG-tagged CHD4 were inserted into pHAGE-3 × FLAG plasmids to generate tagged proteins. In brief, for all virus production, lentiviral vectors and packaging constructs were transfected into 293FT cells by using Lipofectamine 3000 Transfection Reagent (Invitrogen). Supernatants containing lentiviruses were collected at 48–72 h after transfection and then used to infect TNBC cell lines. After 24 h of infection, the infected cells were screened with 2.5 μg/ml puromycin for one week, and the surviving cells were frozen and stored in liquid nitrogen for subsequent experiments. All target sequences for the shRNAs are given in Table 3.

**Table 3:** The shRNA target sequences for indicted genes

	Target sequences(5′-3′)
FBXW7	AACACAAAGCUGGUGUGUGCA
CHD4	GCGGCAGTTCTTTGTGAAATG

### Flow cytometry assay

Cells were collected and washed twice with permeabilization wash buffer and were then resuspended in flow cytometry staining buffer (Bio-Rad), and cell numbers were counted. The dissociated cells were then stained with APC-conjugated anti-CD44 antibody (1:50, Cat# 559942, BD Pharmingen) and FITC-conjugated anti-CD24 antibody (1:50, Cat# 555427, BD Pharmingen) for 30 min at 4 °C. After washing twice in flow cytometry buffer, the cells were analyzed by a FACSCalibur flow cytometer (BD Biosciences). For the ALDEFLUOR Assay, cells were harvested by trypsinization, washed in PBS, labeled with Aldefluor Reagent (StemCell Technologies, Grenoble, France) and incubated at 37 °C for 45 min. Finally, all samples were analyzed by a FACS machine (BD Calibur).

### Sphere formation assays

TNBC cells were dissociated into single cells and seeded in 6-well ultralow attachment plates (Corning, NY, USA) at a density of 1000 cells/well in serum-free stem cell-conditioned medium as described above. After culturing for 7–21 days, spheres with diameters > 50 μm were counted by using ImageJ software.

### Ubiquitination and cycloheximide chase assay

For the in vitro ubiquitination assay. HEK293 cells were grown in a 10 cm dish until they reached 80–90% confluency and then were cotransfected with the indicated plasmids. After 48 h of transfection, the cells were treated with the proteasome inhibitor MG-132 (50 μg/ml) for 6 h before harvesting. Then, total protein was extracted from these treated cells and subjected to immunoprecipitation to detect CHD4 ubiquitination following the same protocol used for Co-IP. For the cycloheximide-chase degradation assay, the transfected cells were grown to a density of approximately 80% and exposed to 10 μg/ml cycloheximide for different incubation times. At the indicated time points, the treated cells were then lysed and used to extract proteins for subsequent Western blot assays as previously described.

### Cell cycle and apoptosis analysis

For cell cycle analysis, cells (1 × 10^7^) were harvested, washed with PBS and fixed in 70% ethanol at 4 °C overnight. After washing three times with PBS, the cells were incubated with 5 μl of RNase A for 1 h and stained with 10 μl of propidium iodide (PI) for 30 min at room temperature in the dark. FSC data were analyzed by using a FACSCalibur flow cytometer (BD). For the apoptosis assay, cells were harvested and double stained with Annexin V and propidium iodide (PI) using an Annexin V-FITC/PI Apoptosis Detection Kit (BestBio, Shanghai, China). Fluorescence was measured using a BD FACScan flow cytometer. FSC data were analyzed using Cell Quest software (BD Biosciences).

### Colony formation assay and migration assay

For the clonogenicity assays, TNBC cells were seeded in a 6-well plate (100 cells/well). After incubation for 2 weeks, colonies were washed with cold 1 × PBS, fixed with 4% paraformaldehyde for 5 min, and stained with 0.1 ml of 0.5% crystal violet for 10 min (Millipore Sigma). After fixation, colonies were washed and air-dried, and the colony numbers were counted with ImageJ software. For the migration assay, approximately 1 × 10^4^ TNBC cells were cultured in the upper chamber containing 200 μl serum-free DMEM, and the lower chamber was filled with 600 μl DMEM containing 10% FBS. After 48 h of incubation, the nonmigrating cells on the upper surface of each filter were carefully removed with a cotton swab, and migrated cells adhering to the bottom were stained with 0.5% crystal violet for 20 min and manually counted in five nonoverlapping fields.

### GST pull-down assay

The full-length cDNA sequence of human CHD4 was obtained by PCR using primers containing restriction enzyme sites for Eco RI and XhoI sites. The CHD4 cDNA was then cloned and inserted into the pGEX-5X-1 glutathione S-transferase (GST) fusion plasmid, which encodes a CHD4-GST fusion protein. The pGEX-5X-1-CHD4 vector was then transformed into competent *Escherichia coli* DH5α for expression. The recombinant GST fusion construct was amplified in bacterial cells, and the GST-CHD4 fusion protein from bacterial cells was purified by using GST-binding agarose resin as previously described. The GST protein was used as a negative control. The eluates were analyzed by Western blot with the indicated antibodies.

### TOP/FOP luciferase reporter assay

For the TOP/FOP-Flash assay. shRNA-expressing cells were transfected with TOP flash or FOP flash reporter plasmids together with CMV-Renilla plasmid. The Renilla reniformis luciferase reporter was selected as an internal control. After 48 h of transfection, the relative luciferase activity was measured by using the Dual-Luciferase Reporter Assay System (Promega). Firefly luciferase activity was normalized to Renilla luciferase activity.

### Immunofluorescence staining (IF)

Two TNBC cell lines were seeded on coverslips in a 24-well dish at 5 × 10^4^ cells/well and further incubated for 24 h. Afterward, the cells were washed three times with cold PBS, fixed in 4% paraformaldehyde for 15 min, permeabilized with 0.1% Triton X-100 in PBS for 15 min at room temperature, and washed three times in PBS. After blocking in 1% BSA for 30 min, the cells were incubated overnight at 4 °C with anti-CHD4 antibody and anti-FBW7 antibody. Subsequently, following several washes in PBS, the coverslips were treated with Alexa 488-conjugated anti-mouse secondary antibody as well as Cy3-conjugated anti-rabbit secondary antibody for a duration of 30 min. Upon repeated washing in PBS, the slides were mounted and sealed using DAPI Staining Solution. Confocal microscopy was used to acquire the images.

### Tumor xenograft experiments

For xenograft models, BALB/c-nu mice aged between 4 and 6 weeks were randomly divided into four distinct groups. They were then subcutaneously injected with various cells as indicated at a concentration of 5 × 10^6^ cells/200 μl (n = 4 per group). Tumor volume was measured once a week: Volume = 1/2 (Length × Width 2). The mice were sacrificed after 6 weeks postinjection, and the weight of the xenograft tumors was recorded. Subsequently, the tumors were fixed in 4% formaldehyde and analyzed by IHC staining with the indicated antibodies. All experimental procedures were approved by the Institutional Animal Care and Use Committee (IACUC) of Zhengzhou University.

### Statistical analysis

All cell experiments were carried out three times, and each group included triplicate samples. Quantitative data are presented as the mean ± SD, and qualitative data are presented as percentages. All statistical analyses were performed using SPSS 17.0 software (SPSS, Chicago, IL, USA) and GraphPad Prism 9 (GraphPad Prism, San Diego, CA, USA). Comparisons between the two groups were performed via Student’s t test, and multiple-group comparisons were performed via ANOVA. A p < 0.05 was considered to indicate statistical significance. GSEA was performed by using GSEA JAVA software (http://software.broadinstitute.org/gsea/index.jsp).

## Results

### FBXW7 is downregulated in TNBC specimens and cell lines and predicts unfavorable survival outcomes in TNBC patients

**Fig. 1:**
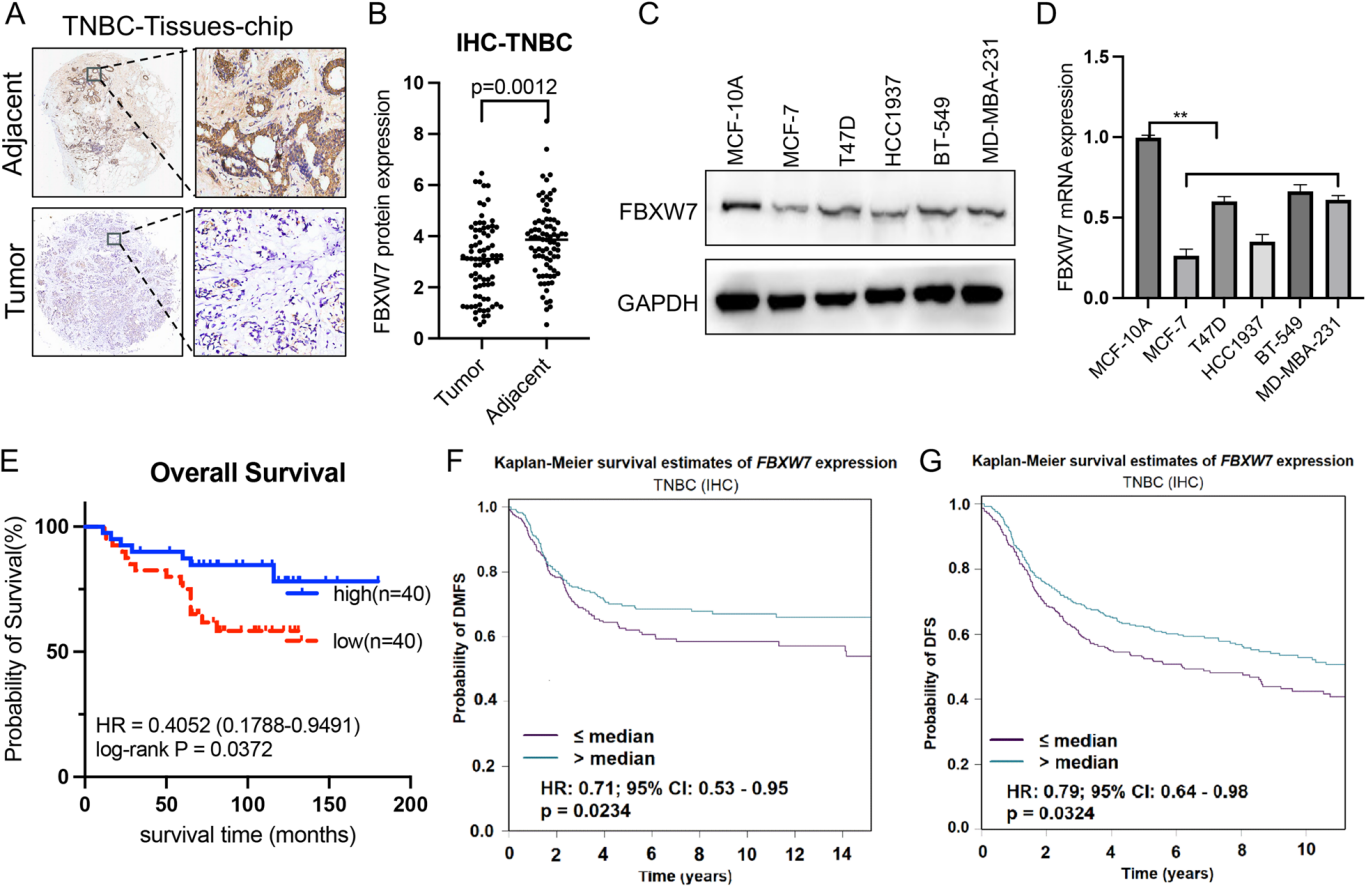
Low expression of FBXW7 in TNBC patients is associated with a poor prognosis. **A** Representative images of immunohistochemical staining of FBXW7 in TNBC tissues and corresponding adjacent noncancerous tissues using TMA tissue sections (n = 80 TNBC). **B** Quantitative values for IHC staining of FBXW7 between tumor tissues and adjacent nontumor normal tissues are presented in the histogram. Statistical analysis was performed by two-tailed paired Student’s t test (p = 0.0012). **C**, **D** Western blot and qRT‒PCR analysis of FBXW7 expression in mammary epithelial cell lines (MCF-10A), two non-TNBC cell lines (MCF-7 and T47-D) and three TNBC cell lines (HCC1937, BT549 and MD-MBA-231). **E** Kaplan–Meier survival curves for OS of TNBC patients in the high FBXW7 and low FBXW7 groups. **F**, **G** Kaplan‒Meier survival curves of DMFS and DFS based on FBXW7 expression using the bc-GenExMiner online tool (http://bcgenex.ico.unicancer.fr/BC-GEM/GEM-Accueil.php?js=1); HR values are shown

Currently, the cancer-related role of FBXW7 in TNBC is unclear. To evaluate the clinical significance of FBXW7 in TNBC, we first performed IHC staining in a tumor tissue microarray comprising 80 pairs of TNBC and adjacent nontumor tissues. Of note, quantification of the data revealed that FBXW7 expression was significantly lower in TNBC specimens than in adjacent noncancerous breast tissues (Fig. 1A and B). Similarly, the protein and mRNA levels of FBXW7 were lower in five BC cell lines than in MCF-10A cells, similar to the results shown in the specimen study (Fig. 1C and D). Subsequently, Kaplan‒Meier curve analysis showed that low expression of FBXW7 was significantly associated with poor overall survival (OS) (Fig. 1E), distant metastasis-free survival (DMFS) (Fig. 1F) and disease-free survival (DFS) (Fig. 1G). Collectively, these data suggested that FBXW7 is significantly downregulated in TNBC and might play a role as a cancer suppressor and favorable prognostic factor in TNBC patients.

### FBXW7 suppresses the malignant phenotype of TNBC cells

**Fig. 2:**
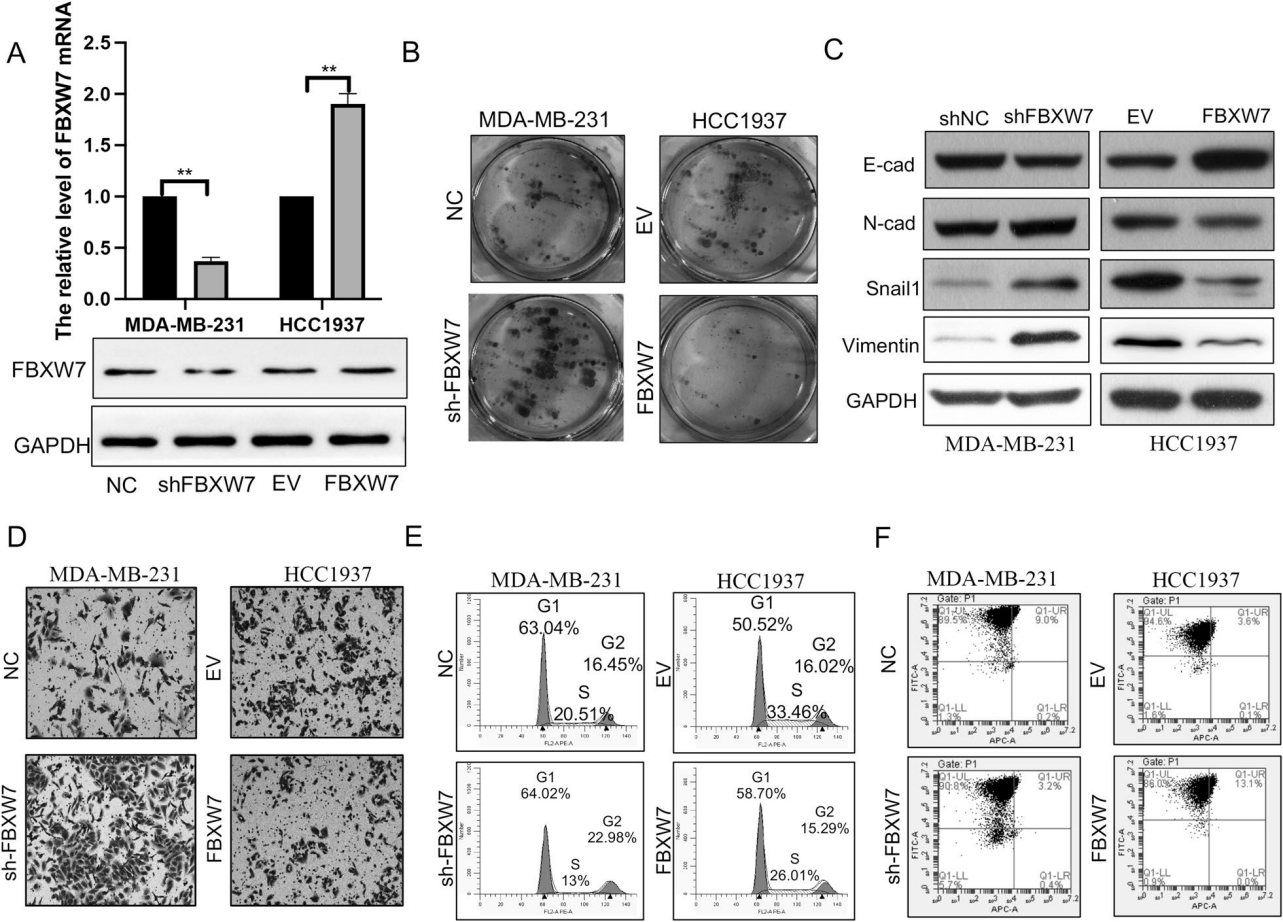
FBXW7 suppresses cell proliferation, cell cycle progression, apoptosis, and EMT in TNBC cells. **A** Western blot and qRT‒PCR analysis of FBXW7 expression following FBXW7 knockdown in MD-MBA-231 cells and FBXW7 overexpression in HCC1937 cells. ***p < 0.0001. **B** Colony formation assays were performed to measure the proliferation ability of the transfected TNBC cells. **C** The levels of EMT marker proteins in the transfected TNBC cells were detected using Western blot analysis. **D** Transwell assays were performed to determine the migration abilities after FBXW7 knockdown and overexpression in MD-MBA-231 and HCC1937 cells, respectively. **E**, **F** FACS assays were performed on the transfected TNBC cells to analyze the cell cycle and apoptosis

To reveal how FBXW7 affects TNBC progression, FBXW7 shRNA and overexpression vector were transfected into the selected TNBC cell lines (MDA-MB-231 and HCC1937, respectively). The silencing and overexpression efficiency of FBXW7 was verified by qRT‒PCR and Western blotting (Fig. 2A). Next, we examined the proliferation abilities of HCC1937 and MDA-MB-231 cells with colony formation assays. Knockdown of FBXW7 significantly promoted the growth of MDA-MB-231 cells. As expected, enhanced FBXW7 expression obviously suppressed the proliferation of HCC1937 cells (Fig. 2B). Western blot assays were further used to verify the role of FBXW7 in the EMT progression of TNBC cells. Upregulating FBXW7 expression observably increased the levels of epithelial markers (E-cadherin) and decreased mesenchymal markers (N-cadherin, Snail1 and Vimentin). In contrast, silencing FBXW7 induced the opposite effects on these EMT markers (Fig. 2C). Transwell assays were performed to evaluate whether interfering with FBXW7 expression can regulate the migration ability of TNBC cells. The results demonstrated that HCC1937 cells with FBXW7 overexpression migrated much more slowly than the control cells, whereas FBXW7 knockdown promoted cell migration in MDA‐MB‐231 cells (Fig. 2D). Then, flow cytometry was used to detect the effect of FBXW7 on the cell cycle and cell apoptosis. We found that forced expression of FBXW7 induced cell apoptosis and cell cycle arrest at the G1 phase in HCC1937 cells, but FBXW7 knockdown reversed the tumor-inhibiting effect of FBXW7 overexpression on the cell cycle and apoptosis in MDA-MB-231 cells (Fig. 2E and F). Quantitative analysis of the above results is shown in Additional file 1: Fig. S1. All these data showed that FBXW7 suppresses oncogenic phenotypes. Collectively, these data provide evidence that FBXW7 serves as a tumor suppressor in modulating the oncogenic phenotypes of TNBC cells.

### FBXW7 maintains the CSC stemness of TNBC in vitro

**Fig. 3:**
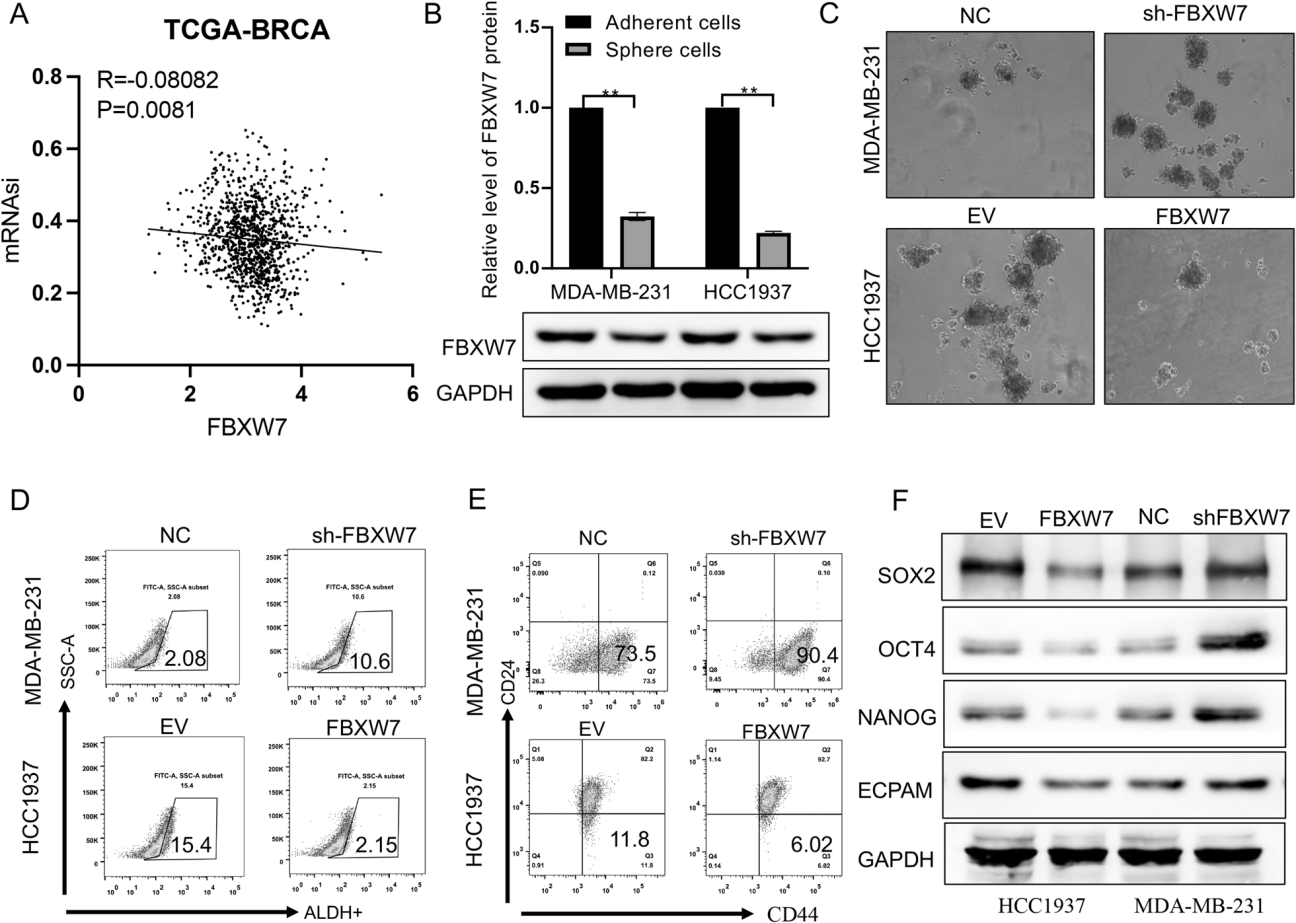
FBXW7 overexpression inhibits the stem cell-like properties of TNBC cells. **A** Pearson’s correlation coefficient analysis of the correlation analysis between FBXW7 and mRNAsi in TCGA-BRCA datasets (r = − 0.08082, p = 0.0081). **B** FBXW7 expression was detected in spheres and their adherent TNBC cells by Western blotting. **C** Representative images and quantitative analysis of spheres. Scale bar: 100 mm. **D** ALDEFLUOR assay of the percentage of ALDH-positive cells in different treatment groups. **E** FACS profiles and quantification of the CD44^high^/CD24^low^ subpopulation. **F** Western blot analysis of the protein levels of stem cell markers (SOX2, OCT4, NANOG and EPCAM) in different cell treatment groups

Our previous studies indicated that FBXW7 negatively regulates cancer stemness in NSCLC cells. In this study, we first found that there was a significant negative correlation between FBXW7 expression and mRNAsi (a stemness index) in TCGA-BC by bioinformatics analysis (Fig. 3A). Then, we enriched CSCs by inducing tumor sphere formation in the TNBC cell lines HCC1937 and MDA-MB-231, measured the mRNA and protein levels of FBXW7, and found that FBXW7 was downregulated in tumor spheres compared to the corresponding adherent cells (Fig. 3B). Afterward, tumor sphere-forming assays were performed to characterize the self-renewal capacity of CSCs. The number and size of tumor spheres were significantly reduced in HCC1937 cells after FBXW7 overexpression and increased greatly in MDA-MB-231 cells after FBXW7 silencing (Fig. 3C). Previous studies identified aldehyde dehydrogenase (ALDH) and CD44^+^CD24^−/low^ as markers of BCSCs in TNBC. These surface markers of BCSCs were assessed by ALDEFLUOR assay and flow cytometry. The results showed that silencing FBXW7 increased the proportion of ALDH-positive cells (from 2.08 to 10.6%) and CD44 + CD24−cells (from 73.5 to 90.4%) among MDA-MB-231 cells. In contrast, the percentage of these cell populations among all cells was significantly reduced by FBXW7 overexpression in HCC1937 cells (Fig. 3D and E). Subsequently, Western blotting was performed to evaluate the effect of FBXW7 on stemness-related genes in TNBC. The WB results proved that enhancing or silencing FBXW7 inhibited or promoted the expression of stemness-related genes such as EPCAM, NANOG, SOX2, and OCT4 in the two TNBC cell lines (Fig. 3F). Quantitative analysis of the above results is shown in Additional file 1: Fig. S2. In short, the results demonstrated that FBXW7 plays a negative role in the maintenance of the stem cell characteristics of TNBC.

### The E3 ubiquitin ligase FBXW7 physically binds to CHD4

**Fig. 4:**
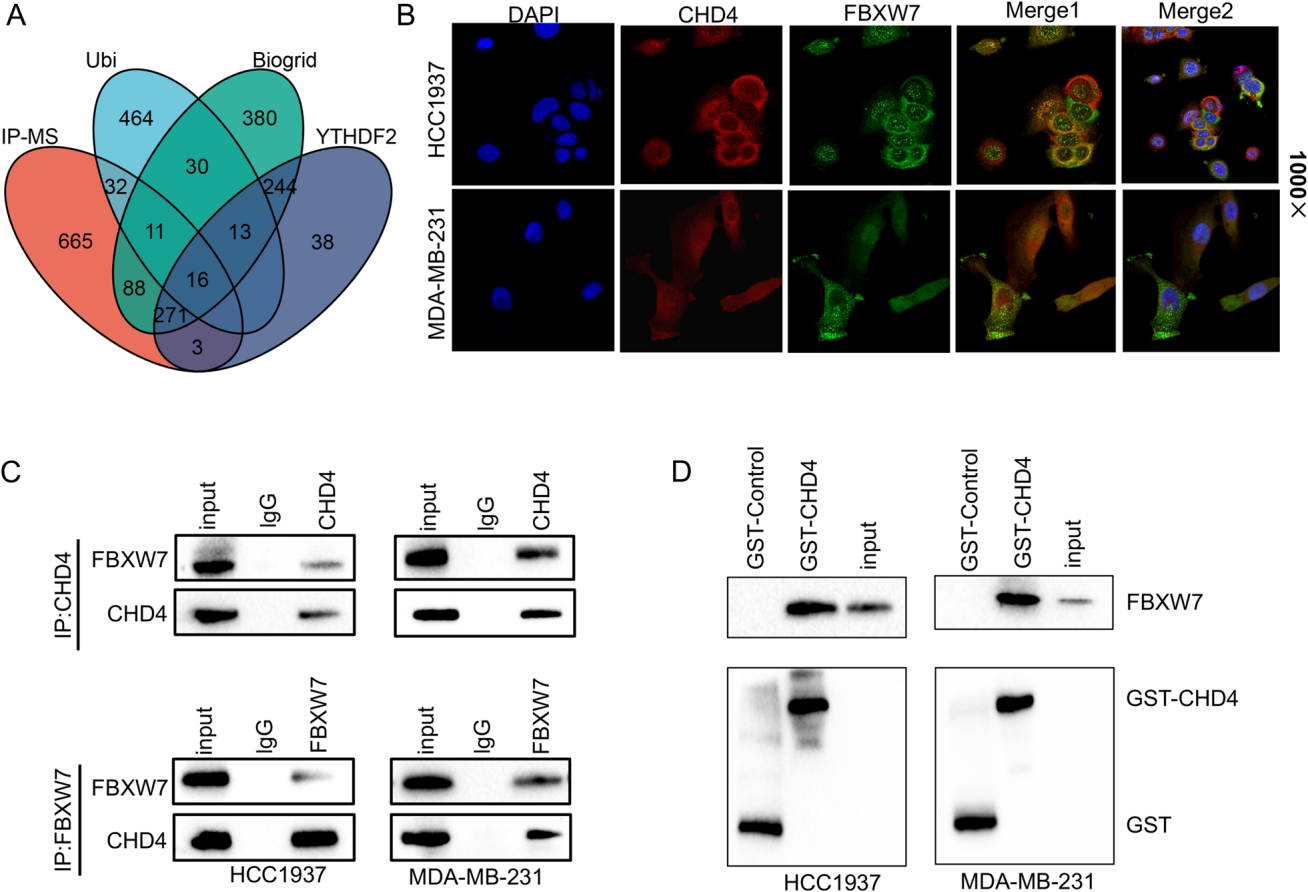
CHD4 is a binding partner of FBXW7. **A** Venn diagram showing the number of overlapping genes between the four datasets as indicated. **B** Intracellular localization analysis of FBXW7 and CHD4 by immunofluorescence assay. The intracellular localization of CHD4 (red) and FBXW7 (green) is shown. Nuclei (blue) were stained with 4′,6-diamidino-2-phenylindole (DAPI). **C** Co-IP assays and Western blotting were conducted to detect the relationship between CHD4 and FBXW7. **D** A GST pull-down assay and Western blotting were performed to determine the interaction between FBXW7 and CHD4

In most malignancies, FBXW7 depends on ubiquitin-mediated degradation of various oncoproteins to exert its tumor-suppressing function. To explore whether FBXW7 also depends on a new substrate to regulate the stemness of TNBC cells, we conducted IP combined with mass spectrometry assays to identify FBW7-interacting proteins. Then, 1615 candidate proteins were obtained from IP-MS analysis (Additional file 2: Table 1), and following intersection with the UbiBrowser database (http://ubibrowser.ncpsb.org.cn), BioGRID database (www. thebiogrid. org) and the published literature [16], 16 candidate proteins were identified (Fig. 4A). We further used the STRING online database to analyze the interactions between FBXW7 and 16 screened proteins and ultimately identified 6 candidate proteins (Additional file 1: Fig. S3). Of these six proteins, CHD4 has been reported in the literature to be more closely associated with cancer stemness. Next, we examined the physical links between FBXW7 and CHD4 in the above cell lines by immunofluorescence (IF) staining and co-IP assays. IF staining experiments confirmed the nuclear colocalization of FBXW7 and CHD4 in the two TNBC cell lines (Fig. 4B). To determine whether endogenous FBXW7 could bind to CHD4, the lysates of two TNBC cell lines were subjected to immunoprecipitation experiments with an anti-FBXW7 antibody and probed for CHD4 with an anti-CHD4 antibody (Fig. 4C). GST pull‐down assay results also confirmed the direct interaction between FBXW7 and CHD4 (Fig. 4D). Overall, the results indicate that FBXW7 can directly bind to CHD4 in TNBC cells.

### FBXW7 decreases CHD4 protein levels and triggers CHD4 degradation via the ubiquitin‐proteasome pathway

**Fig. 5:**
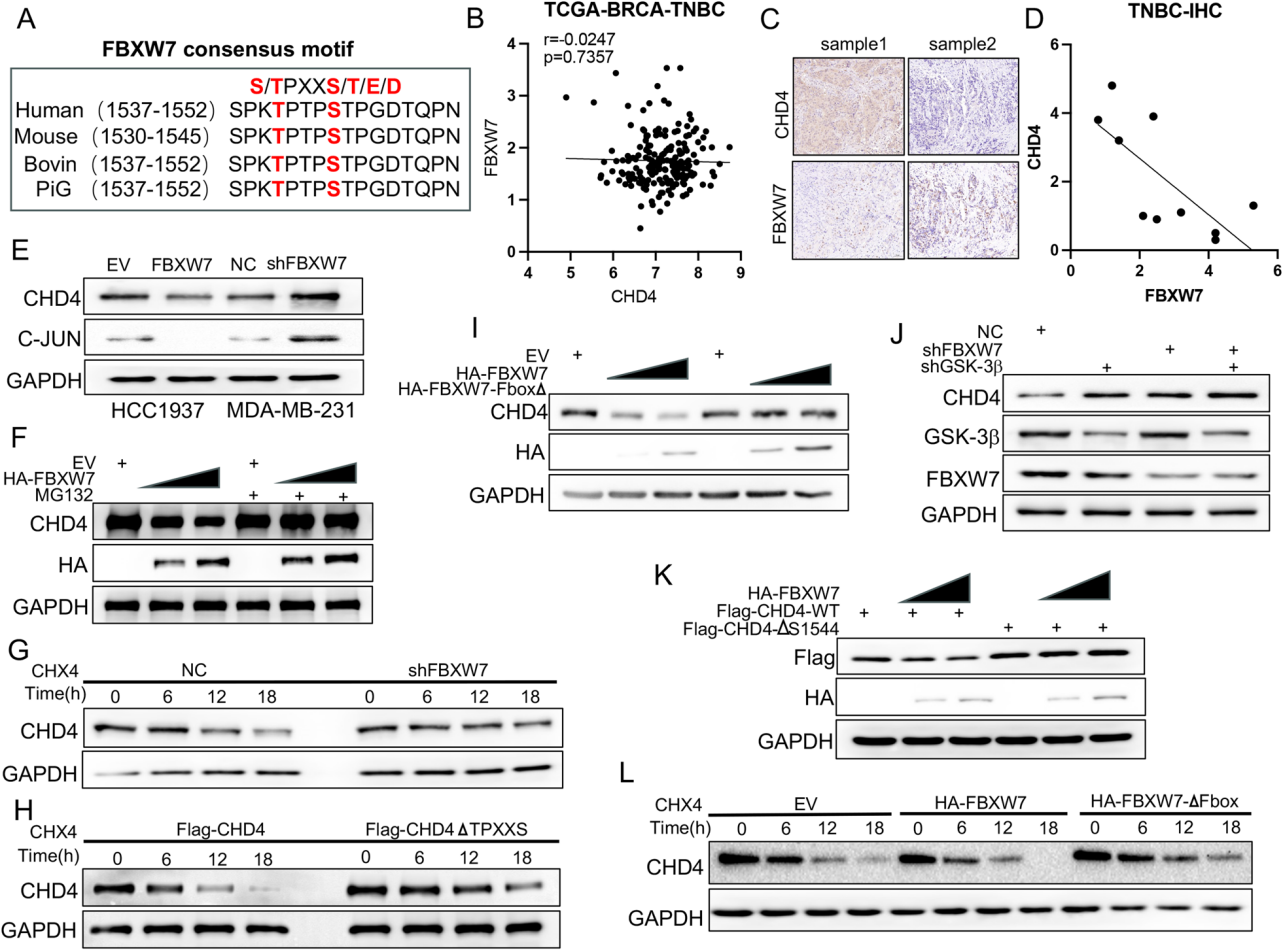
CHD4 protein stability is regulated by FBXW7-mediated ubiquitination. **A** Sequence alignment of the phosphodegron sequences recognized by FBXW7 in CHD4. Conserved degron sequences are shown in red. **B** Scatter diagram showing the correlation between the RNA expression levels of CHD4 and FBW7 in TNBC patients based on TCGA-BRCA-TNBC datasets. P values are indicated. **C** Representative IHC images of FBXW7 and CHD4 staining. The scale bar indicates 50 μm. **D** The correlations between the protein expression of CHD4 and FBXW7 in TNBC tissues. **E** Western blot analysis of the regulation of CHD4 and JUN protein expression by FBXW7 in two transfected TNBC cell lines. **F** Western blot analysis of CHD4 protein and HA-tag protein in HCC1937 cells with different treatments. which was indicated in the figure labels. **G** MDA-MB-231 cells were transfected with shNC or shFBXW7 plasmid and evaluated for CHD4 protein half-life by CHX chase assay. **H** MDA-MB-231 cells were transfected with Flag-CHD4 or Flag-CHD4 ∆TPXXS plasmids and evaluated for CHD4 protein half-life by CHX chase assay. **I** Western blot analysis of CHD4 protein and HA-tag protein in HCC1937 cells transfected with HA-FBXW7 or HA-FBXW7 ∆Fbox plasmid. **J** MDA-MB-231 cells were infected with the indicated constructs for 48 h. Cells were subjected to Western blot analysis. **K** Western blot analysis of Flag-tag protein and HA-tag protein in HCC1937 cells transfected with indicated plasmid. **L** MDA-MB-231 cells were transfected with empty vector, HA-FBXW7 or HA-FBXW7∆Fbox plasmid and evaluated for CHD4 protein half-life by CHX chase assay. GAPDH served as an internal reference in all of the above Western blot analyses

**Fig. 6:**
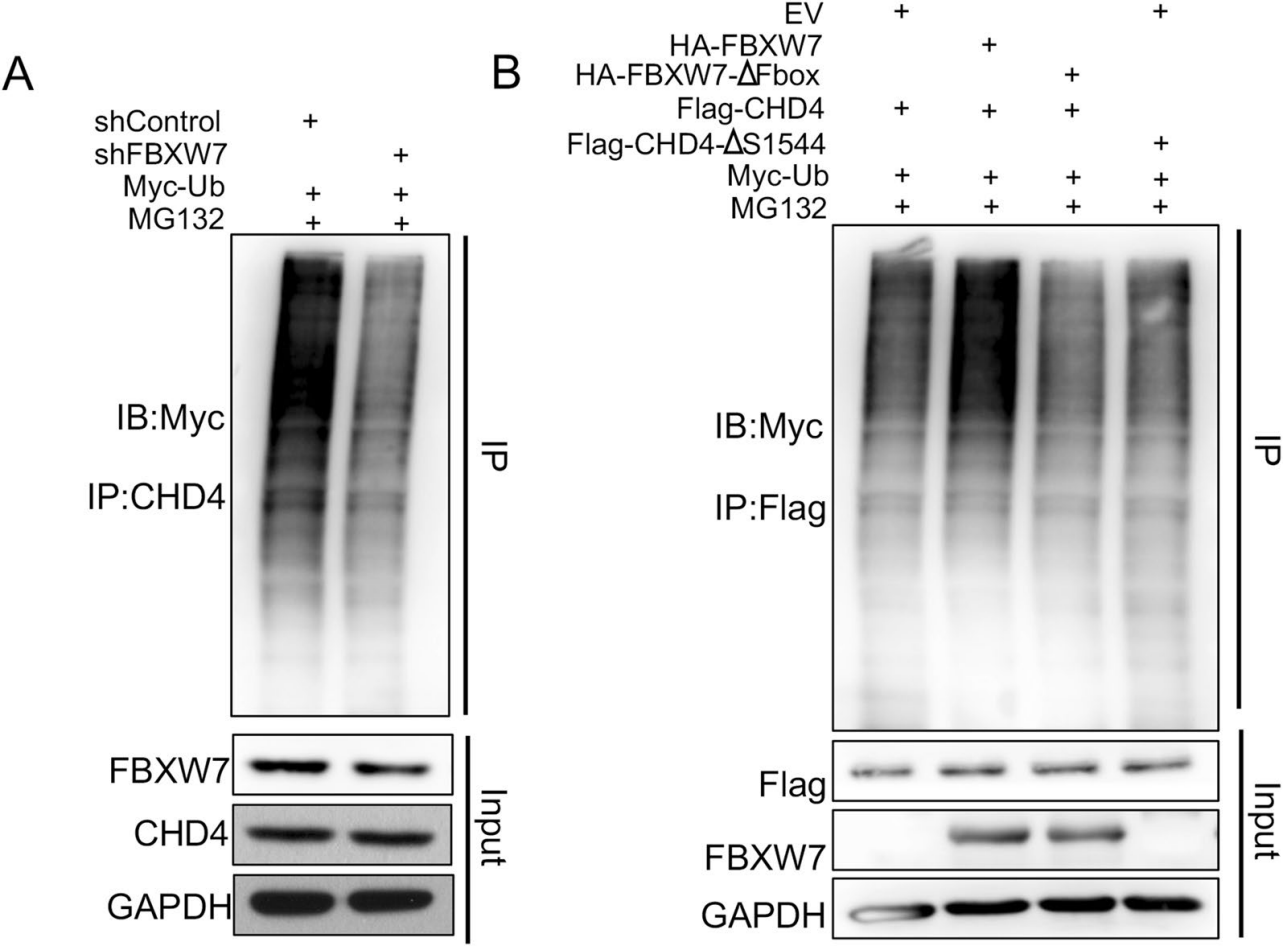
FBW7 serves as an E3 ubiquitin ligase for the CHD4 protein. **A**, **B** Western blot analysis of whole cell lysates (WCLs) and immunoprecipitates derived from HEK-293 T cells transfected with the indicated plasmids for 24 h; cells were treated with 20 μM MG132 for an additional 5 h before harvesting. The ubiquitination status of endogenous CHD4 protein or Flag-tag protein was determined by immunoprecipitation (IP). GAPDH was used as an equal loading control

FBW7 recognizes and binds to a high-affinity recognition motif termed the Cdc4 phosphodegron (CPD) motif (T/S‐P‐X‐X‐S/T/D/E) of its substrates. By using online bioinformatic tools, it was predicted that the CHD4 protein contains one conserved CPD sequence that interacts with FBXW7 phosphate-binding pockets (Fig. 5A). Based on the analysis of samples from the TCGA database, we found that the correlation between FBXW7 and CHD4 at the mRNA level was not significant (Fig. 5B). At the protein level, there was a significant negative correlation between their expression in TNBC samples (Fig. 5C and D). This indicates that the regulation of CHD4 by FBXW7 may occur at the posttranscriptional level. As FBXW7 is an E3 ligase, we first evaluated whether FBXW7 could regulate CHD4 protein stability. As expected, knockdown of FBW7 was found to increase CHD4 protein levels in TNBC cells (Fig. 5A). In contrast, overexpression of FBW7 inhibited CHD4 protein levels, and this effect was diminished by proteasome inhibitor (MG132) treatment (Fig. 5F) or transfection with the F-box-deleted FBW7 mutant (Fig. 5I). Similarly, overexpression of FBXW7 reduced the expression of exogenous CHD4 but did not influence the CHD4 protein with CPD site mutations (Fig. 5K). In addition, the protein half-life of CHD4 was prolonged in FBXW7-KD MDA-MB-231 cells treated with cycloheximide (CHX, an inhibitor of protein synthesis) for the indicated number of hours (Fig. 5G). Likewise, deletion of the CPD sites in CHD4 also delayed the decline in CHD4 protein levels in TNBC cells (Fig. 5H). Significantly, FBXW7 overexpression shortened the protein half-life of endogenous CHD4, but overexpression of its F-box domain–deleted mutant did not induce this effect (Fig. 5L). As most substrates targeted for degradation by FBXW7 require GSK3β-mediated phosphorylation, we examined whether FBXW7 also targets CHD4 in a GSK3β-dependent manner. FBXW7 knockdown partially eliminated the inhibitory effect of GSK3β on CHD4 protein levels (Fig. 5J). Silencing FBXW7 results in decreased polyubiquitination of CHD4, while FBXW7 overexpression increases polyubiquitination of these proteins (Fig. 6A and B). Together, these data suggested that CHD4 might be the substrate of FBW7 in TNBC cells.

### WNT/β‐catenin is a downstream signaling pathway of the FBXW7-CHD4 axis

**Fig. 7:**
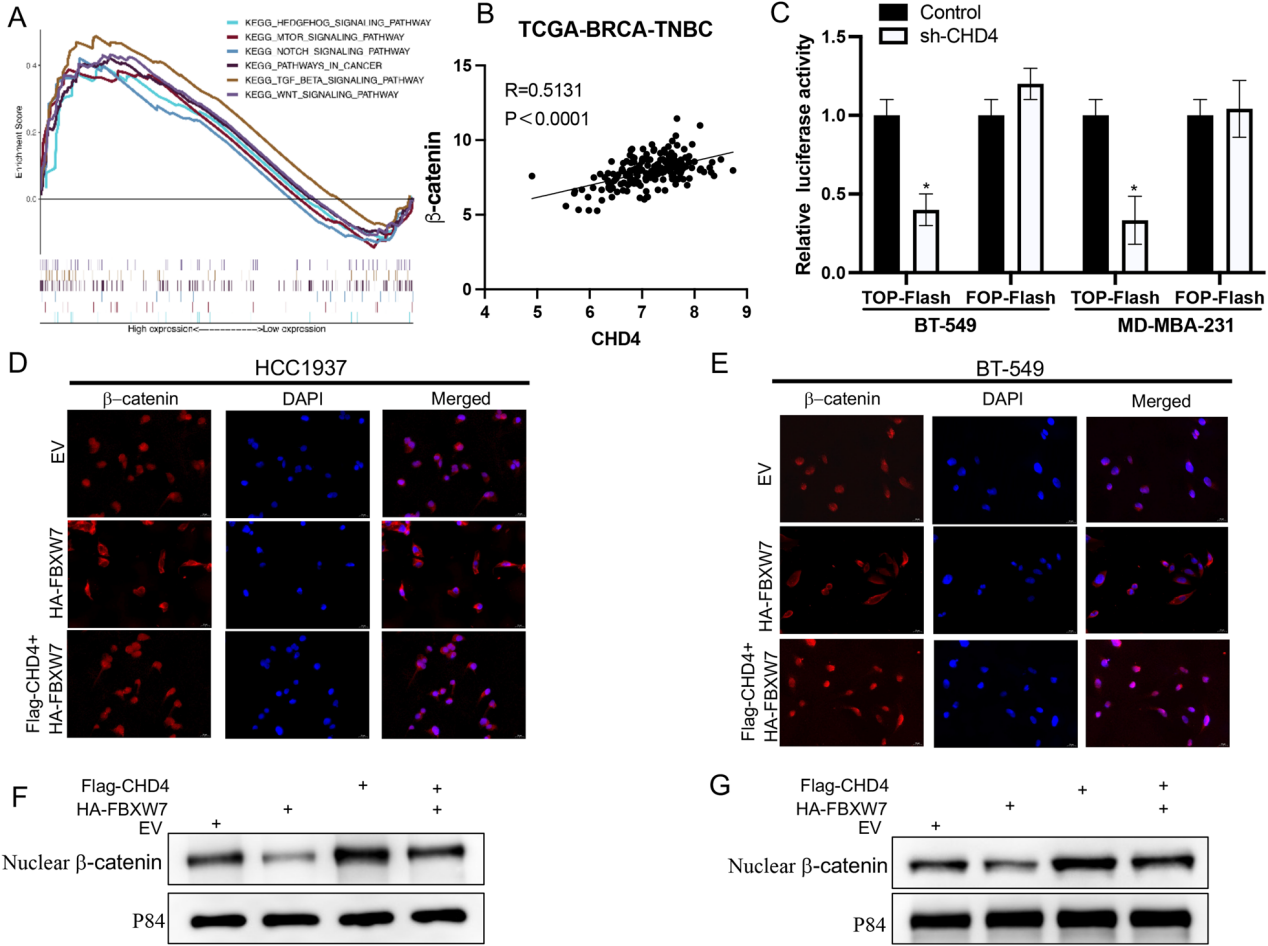
The FBXW7/CHD4 axis regulates the Wnt/β-catenin pathway in TNBC cells. **A** Enrichment plots from GSEA to examine the correlation between CHD4 expression and multiple cancer-related pathways based on TCGA-BRCA-TNBC datasets. **B** Pearson’s correlation analysis of the relationship between β-catenin expression and CHD4 expression was performed. (r = 0.5131, p < 0.0001). **C** The TOP/FOP assay was applied to measure the activity of the Wnt signaling pathway in cells transfected with shControl or shCHD4. **D**, **E** Immunofluorescence assay examining the nuclear translocation of β-catenin upon treatment with the indicated shRNA. Cell nuclei are stained with DAPI dye (blue). β-Catenin fluorescence intensity was analyzed and quantified by ImageJ (right panel) (200 ×). **F**, **G** Western blot analysis was used to examine the β-catenin protein level in the nuclear fraction (nucleus). The quantification of β-catenin protein levels was normalized to that of p84 (nucleus)

Given that CHD4 has been confirmed to be a downstream substrate protein of FBXW7, we aimed to further uncover the underlying molecular signaling pathways involved in the FBXW7-CHD4 axis. Through gene set enrichment analysis (GSEA), we found that cancer-related pathways, including the TGF-beta signaling pathway, Wnt signaling pathway, mTOR signaling pathway, Hedgehog signaling pathway, and Notch signaling pathway, were enriched in TNBC with the CHD4-high phenotype (Fig. 7A). A previous study confirmed that CHD4 promoted β-catenin accumulation in the nucleus of ovarian cancer cells. The results of GSEA and previous studies drew our attention to the Wnt/β-catenin signaling pathway. Nuclear β-catenin accumulation is a hallmark of Wnt/β-catenin pathway activation. Based on bioinformatics analysis, we found that CHD4 positively correlated with β-catenin in TNBC samples (Fig. 7B). Then, TOP/FOP luciferase reporter assays were performed to detect β-catenin activity, and the results revealed that knockdown of CHD4 inhibited TOP/FOP luciferase activity (Fig. 7C). Immunofluorescence assays and quantitative analysis confirmed that FBXW7 overexpression significantly decreased the amount of β-catenin in the nucleus compared with that in the control group, and this inhibitory effect was reversed by enforced CHD4 expression in the two TNBC cell lines (Fig. 7D and E). Further Western blot experiments confirmed that the FBXW7-CHD4 axis regulates the accumulation of β-catenin in the nucleus (Fig. 7F and G). Quantitative analysis of the above results is shown in Additional file 1: Fig. S4.

### The FBXW7-CHD4-WNT/β‐catenin axis is needed for the maintenance of TNBC stemness

**Fig. 8:**
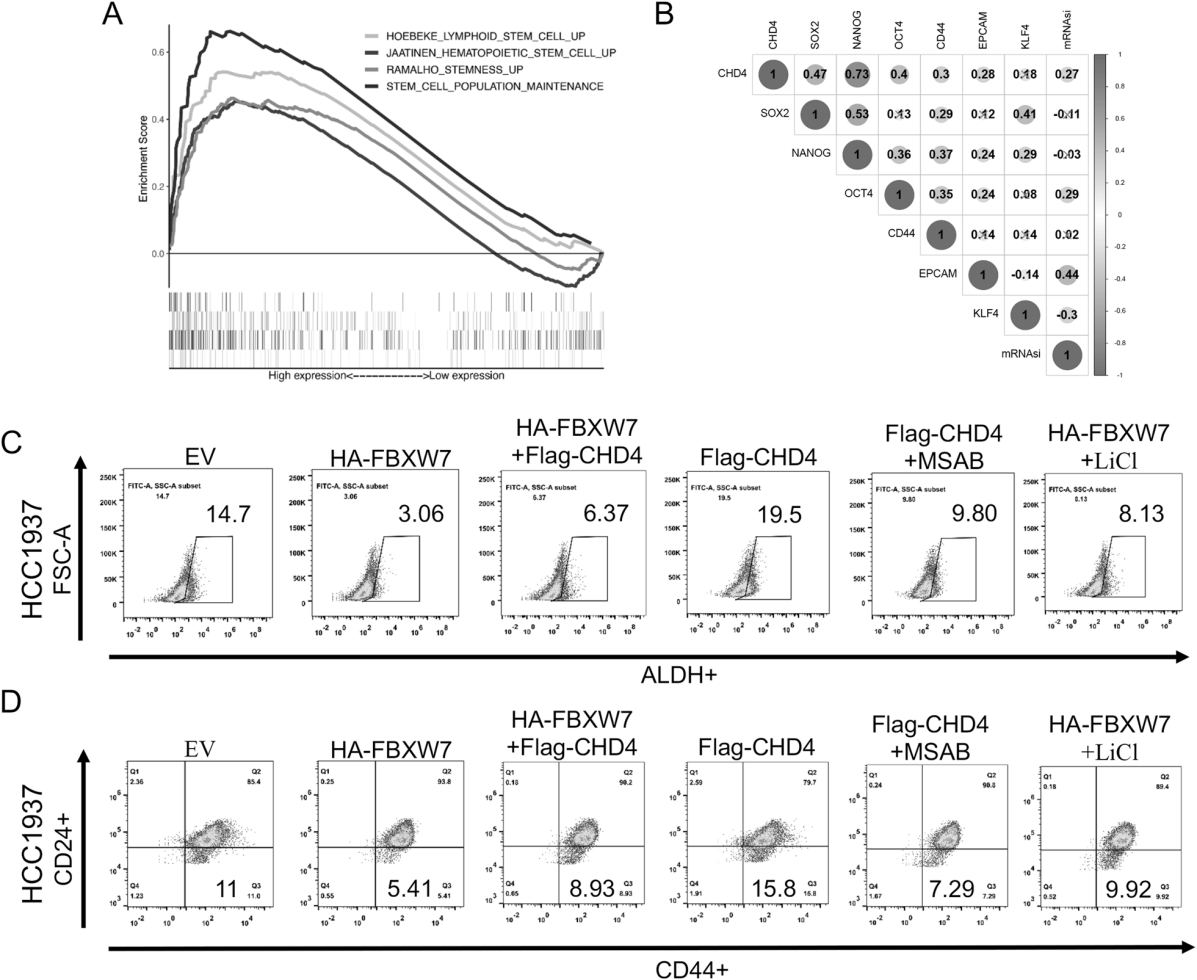
The FBXW7/CHD4/Wnt/β-catenin axis regulates the stem cell-like properties of TNBC cells. **A** Enrichment plots from GSEA to examine the correlation between CHD4 expression and multiple stemness-related gene sets based on TCGA-BRCA-TNBC datasets. **B** Heatmap analysis of the correlation between CHD4 expression and multiple stemness-related genes based on TCGA-BRCA-TNBC datasets. **C** ALDEFLUOR assay of the percentage of ALDH-positive cells in different treatment groups. **D** FACS profiles and quantification of the CD44^high^/CD24^low^ subpopulation in different treatment groups

Previous reports combined with our results have confirmed that both FBXW7 and Wnt signaling pathways are involved in the stemness regulation of TNBC cells, but it remains unclear whether CHD4 also regulates cancer stem cells in TNBC. GSEA showed that CHD4 was positively associated with multiple stemness- or stem cell-related gene sets in the TCGA-TNBC dataset (Fig. 8A). Correlation analysis suggested that CHD4 was significantly positively correlated with multiple stemness-related genes (SOX2, NANOG, OCT4, CD44 and EPCAM) and a stemness index (mRNAsi) (Fig. 8B). To further evaluate whether the FBXW7-CHD4 axis affects the proportion of BCSCs in TNBC via the Wnt/β-catenin signaling pathway, transfection experiments were performed. Transfection with HA-FBXW7 or Flag-CHD4 significantly decreased or increased the proportions of ALDH + and CD44 + /CD24−BCSCs in HCC1937 cells, respectively, and these effects could be rescued by an activator (LiCl) or inhibitor (MSAB) of Wnt/β-catenin signaling (Fig. 8C and D). Quantitative analysis of the above results is shown in Additional file 1: Fig. S5.

### FBXW7 inhibits tumorigenesis in vivo

**Fig. 9:**
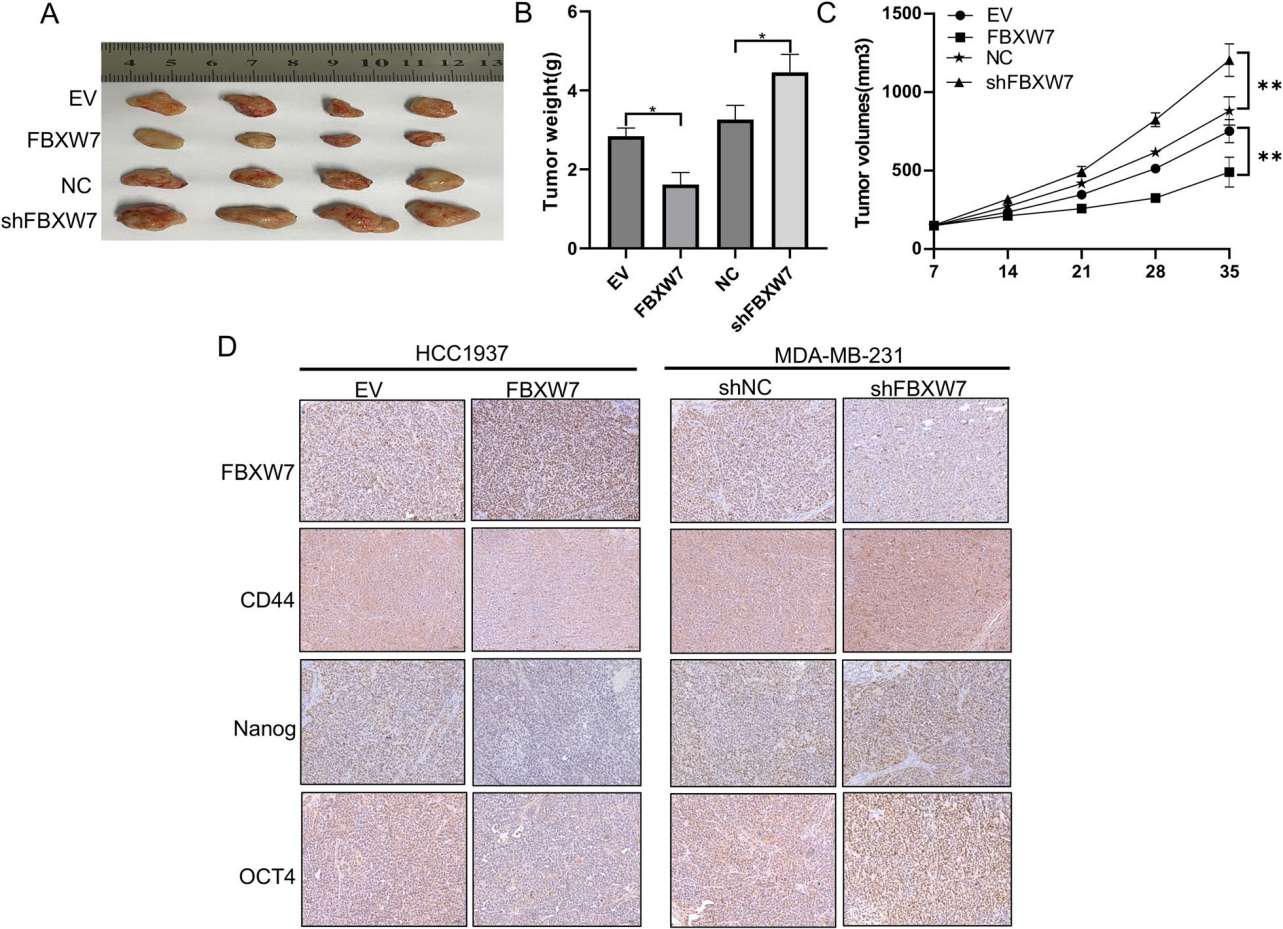
FBXW7 inhibits tumorigenesis in vivo. **A** Representative images of xenografts in nude mice established via subcutaneous injection of TNBC cells. **B**, **C** Growth curve and histogram analysis of the volumes and weights of xenograft tumors. **p < 0.001. **D** Representative IHC staining images of FBXW7, CD44, Nanog and OCT4 in xenograft tumor tissues

To extend our in vitro observations, we investigated the effects of FBXW7 on the tumorigenic potential of TNBC cells in vivo by using a nude mouse model. The results suggested that FBXW7 overexpression significantly inhibited tumor growth and decreased tumor size and tumor weight compared with that in the vector control group, while the opposite trends were observed following FBXW7 knockdown (Fig. 9A–C). Consistently, IHC indicated that the expression of stemness markers (CD44, Nanog, and OCT4) was lower in FBXW7-overexpressing xenograft tumors than in vector group tumors, whereas xenograft tumors grown from FBXW7 knockdown cells expressed higher levels of these markers than tumors grown from control cells (Fig. 9D). This finding confirmed that FBXW7 reduces CSC marker expression and suppresses TNBC cell tumorigenesis in vivo.

## Discussion

Despite the application of multimodal treatments, TNBC patients still have the worst prognosis of all subtypes of breast cancer. Previous studies have proposed that breast cancer stem cells (BCSCs) are enriched in TNBC [17]. BCSCs are a small subpopulation of cells in breast cancer tissues with self-renewal ability, multilineage differentiation potential and high tumorigenicity. Due to these biological properties, BCSCs are often enriched in residual cancer tissue after breast cancer treatment with radiotherapy and chemotherapy [18]. The resistance of BCSCs to conventional anticancer treatments has been considered responsible for recurrence and metastasis. There is substantial evidence to suggest that complete tumor eradication depends on the complete elimination of CSCs, but effectively identifying and targeting CSCs is not simple in cancer therapy. Thus, the precise molecular mechanisms underlying BCSC regulation in TNBC remain largely unknown.

FBXW7 mutations have been frequently observed in various cancer types. FBXW7 primarily targets oncoproteins for degradation and is thus widely recognized as a classic tumor suppressor gene. Previous studies on breast cancer have shown that the mutation frequency of FBXW7 in TNBC is considerably higher than that in ER receptor-positive BC [19]. It has also been observed that FBXW7 plays a role in ubiquitinating and degrading EglN2, thereby inhibiting TNBC tumorigenesis [20]. Additionally, a separate article reported that the circRNA produced by the FBXW7 gene hinders the malignant progression of TNBC cells through the ceRNA mechanism [21]. Until now, only a few studies have explored the role of FBXW7 in the regulation of CSCs in several cancers, such as colorectal CSCs [22], lung CSCs [23], and live CSCs [24]. However, the precise function and mechanism of FBXW7 in the CSC-like properties of TNBC cells remain largely unclear. Hence, this study mainly focused on the stemness of TNBC. In this study, we applied IHC staining to a tissue microarray and revealed that FBXW7 has markedly lower expression in tumor samples. Furthermore, clinical analysis revealed that downregulation of FBXW7 is associated with a poor prognosis in TNBC patients. This study aimed to characterize the functional role of FBXW7 in the stemness of TNBC. As expected, we confirmed that the level of FBXW7 was lower in tumor spheres than in adherent cells. The following functional experiments further validated that FBXW7 suppresses cell proliferation, migration, invasion, self-renewal, and tumorigenicity in TNBC. Thus, we speculated that FBXW7 functions as an inhibitory factor and regulates the stemness of TNBC. These findings may lay the foundation for further unraveling the molecular mechanism of FBXW7 in regulating CSCs in TNBC. To further elucidate the functional targets of FBXW7, we immunoprecipitated epitope-tagged FBXW7 and identified coprecipitating proteins using mass spectrometry. After intersecting these IP-MS proteins with multiple databases, CHD4 was finally screened out as a potential substrate protein for subsequent experiments. In vitro experiments further confirmed that CHD4 physically binds to FBXW7 and that its protein stability can be regulated by FBXW7-mediated ubiquitination and degradation. CHD4 plays a key role in regulating diverse cellular functions under physiological and pathological conditions, especially in carcinogenesis. Accumulating evidence has revealed that CHD4 is highly expressed in TNBC tissues and significantly positively correlated with tumor metastasis status, tumor recurrence, and poor prognosis [15]. More recently, CHD4 was found to mediate EMT in TNBC cells, and silencing CHD4 expression in these cells increased drug sensitivity to cisplatin and PARP1 inhibitors. However, it remains unclear whether CHD4 regulates the stemness of TNBC cells. Interestingly, the regulation of cancer stemness by CHD4 in different malignant tumors is also controversial. Loss of CHD4 function promotes endometrial cancer stemness by activating the TGF-β pathway [14] while suppressing stemness maintenance in papillary thyroid carcinoma [13]. In our study, we used GSEA to find that stemness-related gene sets were significantly enriched in TNBC with high CHD4 gene expression. Correlation analysis also showed that CHD4 was significantly positively correlated with multiple stemness-related genes. Flow cytometry analysis further confirmed that CHD4 maintains the proportion of BCSCs in TNBC cells and partially reverses the inhibitory effect of FBXW7 on BCSCs. These results indicate that FBXW7-mediated degradation of CHD4 is a novel regulatory mechanism of cancer stemness in TNBC.

Wnt/β-catenin signaling is an evolutionarily conserved signaling pathway that is mainly responsible for embryonic development and tissue homeostasis [25]. Growing evidence indicates that Wnt/β-catenin signaling plays a crucial role in maintaining stemness in TNBC [26]. Strikingly, an early study mentioned that CHD4 promoted the nuclear accumulation of β-catenin in ovarian cancer [27]. Consistent with the above reports, we also found that CHD4 was significantly positively correlated with β-catenin in TNBC tissues and promoted β-catenin nuclear accumulation in TNBC cells. Nuclear β-catenin accumulation is a hallmark of Wnt/β-catenin pathway activation. On the other hand, some studies have demonstrated that FBXW7 can directly ubiquitinate and degrade β-catenin to decrease the activity of the Wnt pathway [28]. Moreover, our study confirmed that activating or inhibiting the Wnt pathway can inhibit the effects of FBXW7 and CHD4 on the proportion of BCSCs in TNBC cells. Therefore, we speculate that the FBXW7-CHD4-Wnt/β-catenin signaling axis plays a key role in regulating the stemness of TNBC.

Although the observations are interesting, there are still some limitations and shortcomings to this study. First, we found that GSK-3β knockdown can partially reverse the inhibitory effect of FBXW7 on the CHD4 protein, but we did not further analyze whether GSK phosphorylates CHD4 and clarify the phosphorylation site. Drug resistance is considered a characteristic of tumor-initiating stem cells. Considering that CHD4 knockdown sensitized TNBC cells to cisplatin and PARP inhibitors, it is necessary to further analyze the relationship between the “FBXW7-CHD4-Wnt/β-catenin” signaling axis and chemotherapy resistance in subsequent studies. Given that CHD4 serves as an epigenetic regulator, in future studies, we will further explore whether FBXW7 can also affect epigenetic modifications by degrading CHD4.

## Conclusions

FBXW7 is downregulated in TNBC tissues, and its low expression is closely related to poor prognosis of patients. In vitro studies revealed that FBXW7 inhibits malignant phenotypes and stemness characteristics of TNBC cells. Moreover, CHD4 was identified as a new substrate of FBXW7 in the regulation of cancer stemness in TNBC. Through mechanistic exploration, we confirmed that the FBXW7-CHD4 signaling axis affects the activity of the Wnt/β-catenin pathway by mediating β-catenin nuclear translocation. Therefore, targeting the FBXW7-CHD4-Wnt/β-catenin signaling axis to reduce BCSC stemness may be a potential therapeutic strategy for TNBC.

### Supplementary Information

**Additional file 1: Figure S1.** Statistical analysis of cell proliferation (**A**), cell cycle distribution (**B**, **C**), apoptosis (**D**, **E**) and migration (**F**, **G**) experiments in two TNBC cell lines. **Figure S2.** Statistical analysis of sphere formation (**A**) and the proportion of BCSCs (**B**, **C**) in two TNBC cell lines. **Figure S3.** PPI network of FBXW7 and 16 candidate proteins constructed via the STRING online database. **Figure S4.** Quantitative analysis of nuclear β-catenin based on immunofluorescence results. **Figure S5.** Statistical analysis of the proportion of BCSCs in TNBC cells after transfection with plasmids as indicated.

**Additional file 2**: The list differentially identified proteins by the co-IP-mass spectrometry.

## Data Availability

The datasets used and/or analyzed during the current study are available from the corresponding author on reasonable request.

## Abbreviations

BC: Breast cancer
TNBC: Triple-negative breast cancer
FBXW7: F-box and WD repeat domain containing 7
CHD4: Chromodomain helicase DNA binding protein 4
EMT: Epithelial-mesenchymal transition
IP-MS: Immunoprecipitation-mass spectrometry
EPCAM: Epithelial cell adhesion molecule
qRT‒PCR: Real-time quantitative reverse transcription PCR
mRNAsi: The stemness index based on mRNA expression
BCSCs: Breast cancer stem cells
