# Supplementary figures and images for: Fbxw7 suppresses carcinogenesis and stemness in triple-negative breast cancer through CHD4 degradation and Wnt/β-catenin pathway inhibition
Source: J Transl Med. 2024 Jan 24;22:99. doi: 10.1186/s12967-024-04897-2 (PMC10809768; doi:10.1186/s12967-024-04897-2)

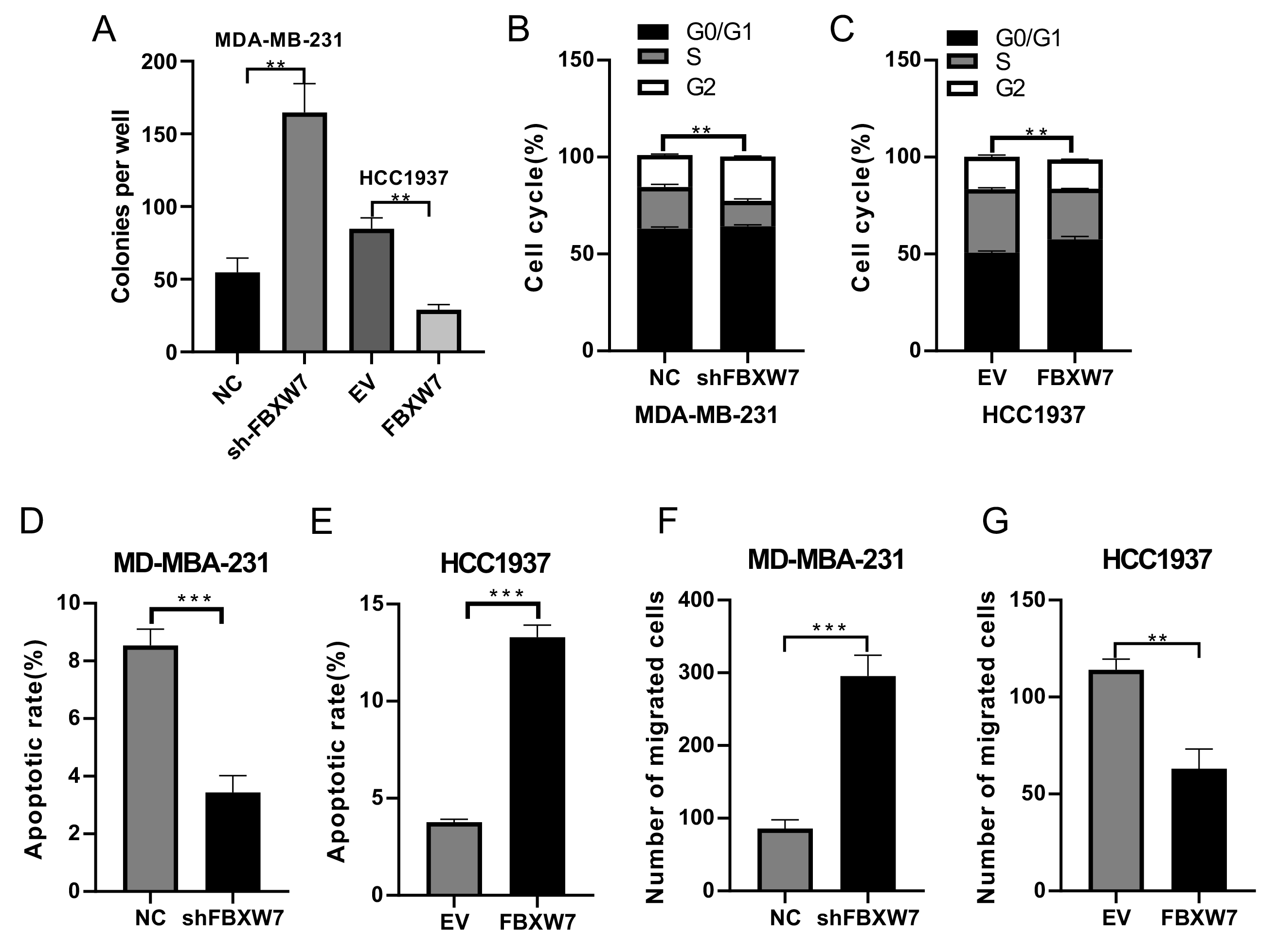

Supplement: Supplementary file 1 — Additional file 1: Figure S1. Statistical analysis of cell proliferation (A), cell cycle distribution (B, C), apoptosis (D, E) and migration (F, G) experiments in two TNBC cell lines. Figure S2. Statistical analysis of sphere formation (A) and the proportion of BCSCs (B, C) in two TNBC cell lines. Figure S3. PPI network of FBXW7 and 16 candidate proteins constructed via the STRING online database. Figure S4. Quantitative analysis of nuclear β-catenin based on immunofluorescence results. Figure S5. Statistical analysis of the proportion of BCSCs in TNBC cells after transfection with plasmids as indicated. [file 12967_2024_4897_MOESM1_ESM.zip › Supplementary/Supplemental Figures1.tif]

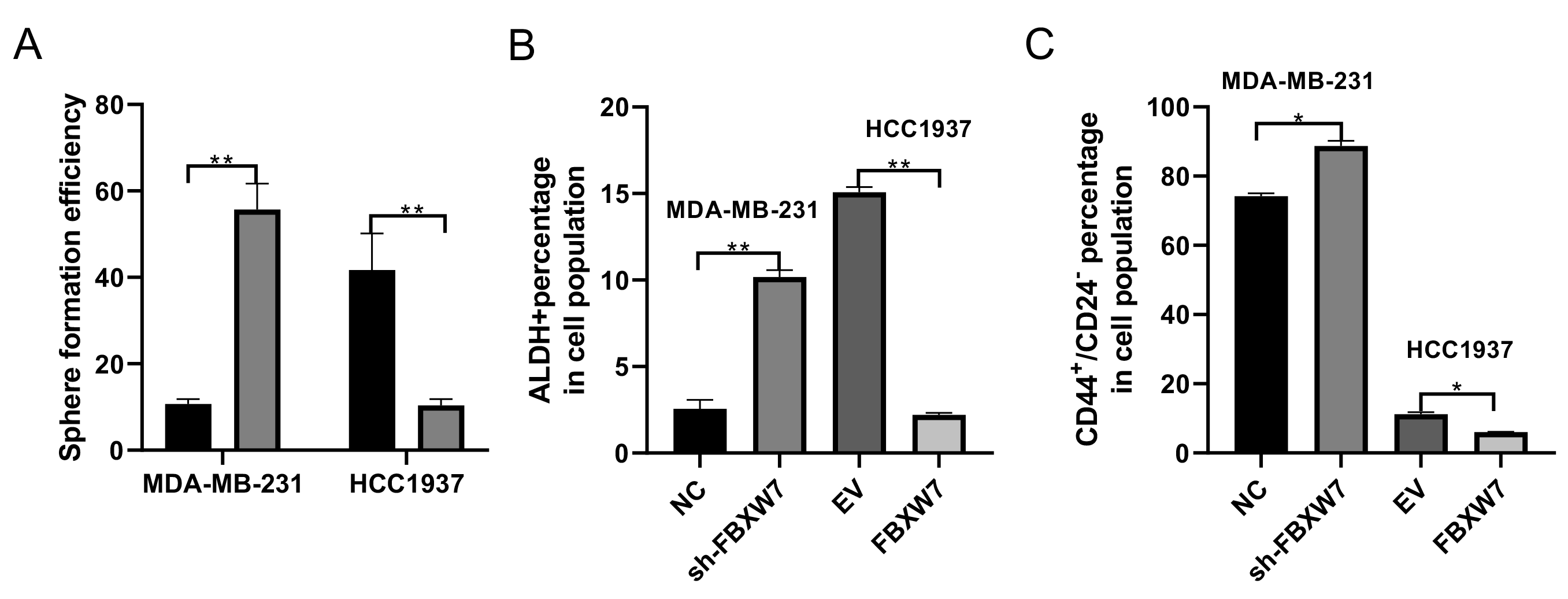

Supplement: Supplementary file 1 — Additional file 1: Figure S1. Statistical analysis of cell proliferation (A), cell cycle distribution (B, C), apoptosis (D, E) and migration (F, G) experiments in two TNBC cell lines. Figure S2. Statistical analysis of sphere formation (A) and the proportion of BCSCs (B, C) in two TNBC cell lines. Figure S3. PPI network of FBXW7 and 16 candidate proteins constructed via the STRING online database. Figure S4. Quantitative analysis of nuclear β-catenin based on immunofluorescence results. Figure S5. Statistical analysis of the proportion of BCSCs in TNBC cells after transfection with plasmids as indicated. [file 12967_2024_4897_MOESM1_ESM.zip › Supplementary/Supplemental Figures2.tif]

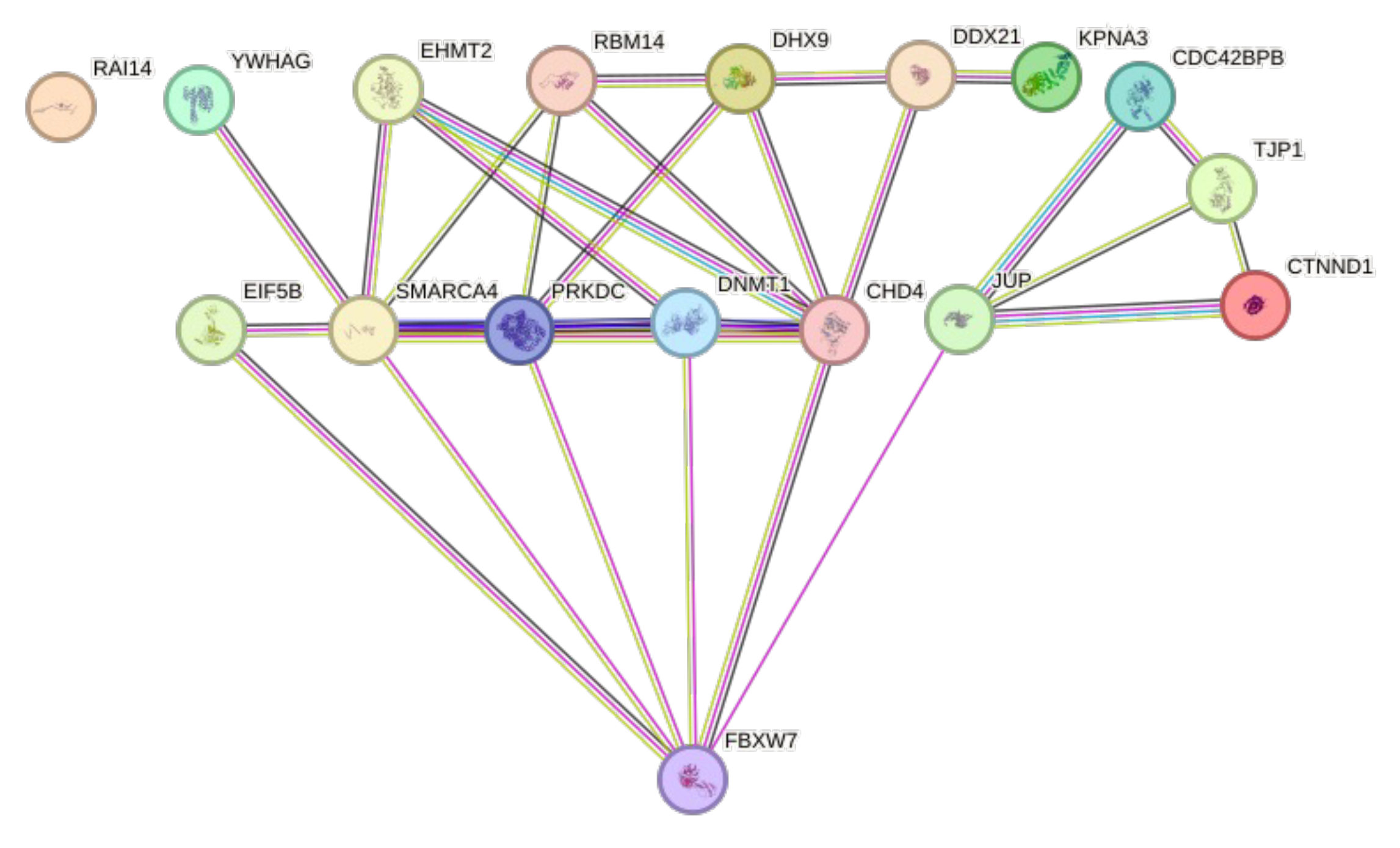

Supplement: Supplementary file 1 — Additional file 1: Figure S1. Statistical analysis of cell proliferation (A), cell cycle distribution (B, C), apoptosis (D, E) and migration (F, G) experiments in two TNBC cell lines. Figure S2. Statistical analysis of sphere formation (A) and the proportion of BCSCs (B, C) in two TNBC cell lines. Figure S3. PPI network of FBXW7 and 16 candidate proteins constructed via the STRING online database. Figure S4. Quantitative analysis of nuclear β-catenin based on immunofluorescence results. Figure S5. Statistical analysis of the proportion of BCSCs in TNBC cells after transfection with plasmids as indicated. [file 12967_2024_4897_MOESM1_ESM.zip › Supplementary/Supplemental Figures3.tif]

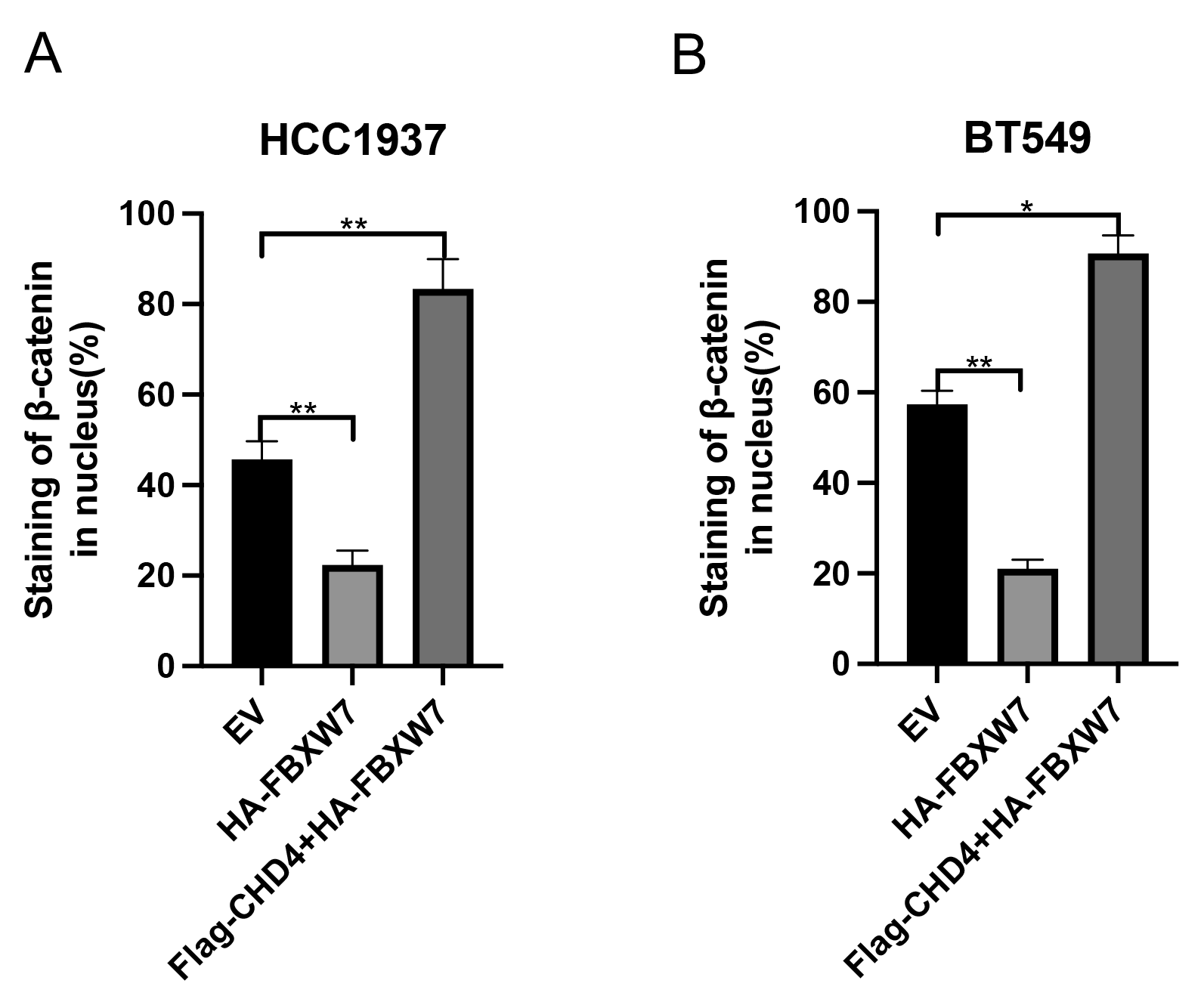

Supplement: Supplementary file 1 — Additional file 1: Figure S1. Statistical analysis of cell proliferation (A), cell cycle distribution (B, C), apoptosis (D, E) and migration (F, G) experiments in two TNBC cell lines. Figure S2. Statistical analysis of sphere formation (A) and the proportion of BCSCs (B, C) in two TNBC cell lines. Figure S3. PPI network of FBXW7 and 16 candidate proteins constructed via the STRING online database. Figure S4. Quantitative analysis of nuclear β-catenin based on immunofluorescence results. Figure S5. Statistical analysis of the proportion of BCSCs in TNBC cells after transfection with plasmids as indicated. [file 12967_2024_4897_MOESM1_ESM.zip › Supplementary/Supplemental Figures4.tif]

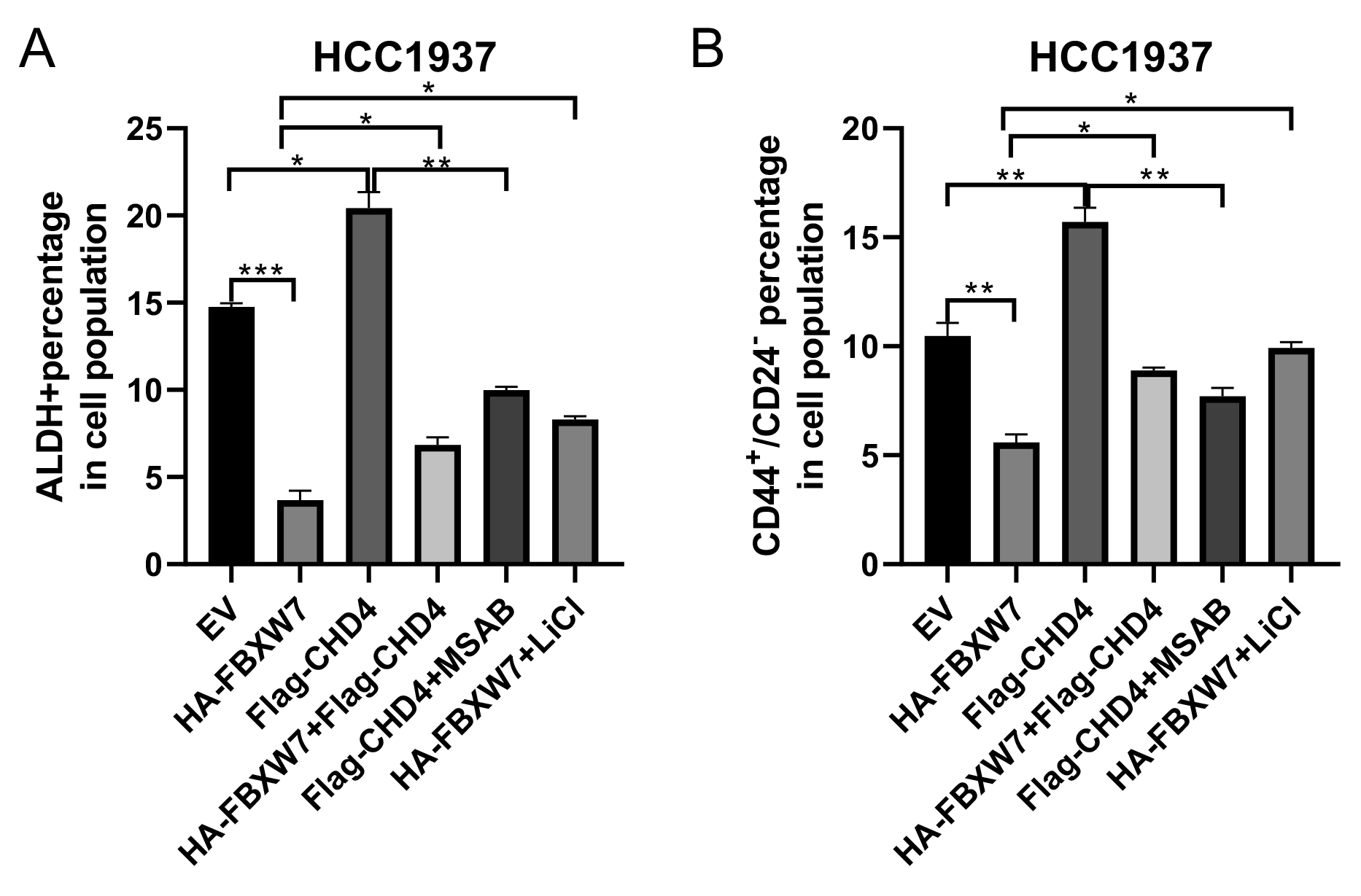

Supplement: Supplementary file 1 — Additional file 1: Figure S1. Statistical analysis of cell proliferation (A), cell cycle distribution (B, C), apoptosis (D, E) and migration (F, G) experiments in two TNBC cell lines. Figure S2. Statistical analysis of sphere formation (A) and the proportion of BCSCs (B, C) in two TNBC cell lines. Figure S3. PPI network of FBXW7 and 16 candidate proteins constructed via the STRING online database. Figure S4. Quantitative analysis of nuclear β-catenin based on immunofluorescence results. Figure S5. Statistical analysis of the proportion of BCSCs in TNBC cells after transfection with plasmids as indicated. [file 12967_2024_4897_MOESM1_ESM.zip › Supplementary/Supplemental Figures5.tif]
